# Disease prediction via Bayesian hyperparameter optimization and ensemble learning

**DOI:** 10.1186/s13104-020-05050-0

**Published:** 2020-04-10

**Authors:** Liyuan Gao, Yongmei Ding

**Affiliations:** College of Science, Wuhan University of Science and Technology, Huangjiahu West Road, Wuhan, 430065 China

**Keywords:** Hyperparameter optimization, Feature selection, Ensemble learning, Gain

## Abstract

**Objective:**

Early disease screening and diagnosis are important for improving patient survival. Thus, identifying early predictive features of disease is necessary. This paper presents a comprehensive comparative analysis of different Machine Learning (ML) systems and reports the standard deviation of the results obtained through sampling with replacement. The research emphasises on: (a) to analyze and compare ML strategies used to predict Breast Cancer (BC) and Cardiovascular Disease (CVD) and (b) to use feature importance ranking to identify early high-risk features.

**Results:**

The Bayesian hyperparameter optimization method was more stable than the grid search and random search methods. In a BC diagnosis dataset, the Extreme Gradient Boosting (XGBoost) model had an accuracy of 94.74% and a sensitivity of 93.69%. The mean value of the cell nucleus in the Fine Needle Puncture (FNA) digital image of breast lump was identified as the most important predictive feature for BC. In a CVD dataset, the XGBoost model had an accuracy of 73.50% and a sensitivity of 69.54%. Systolic blood pressure was identified as the most important feature for CVD prediction.

## Introduction

Modern medical methods prevent disease through early intervention rather than treatment after diagnosis. Early screening and detection of diseases are major issues in the field of healthcare. Breast cancer and cardiovascular Disease are the most common diseases among women and elderly people, respectively [1–3]. Globally, approximately 1.3 million new cases of BC are reported each year. BC has the highest incidence in developed countries, but it has also increased at an alarming rate in low- and middle-income countries [4]. In addition, CVD accounts for approximately half of all deaths in most European countries [5]. Early screening and diagnosis of BC and CVD are the most effective ways to detect early disease and reduce mortality [6, 7]. The prediction of BC diagnosis through Logistic Regression (LR) and cross-validation results in a prediction accuracy of 96.2%, providing a basis for computer system diagnosis of breast cytology [8]. The current machine learning algorithms for BC and CVD prediction are mainly focused on Support Vector Machine (SVM), Neural Networks (NNs), and Decision Tree (DT) models. In analyses of BC diagnosis datasets, Random Forest (RF) [9] and SVM [10] have achieved better prediction results than other algorithms. In particular, kernel-based SVM can achieve a classification accuracy of 83.68% [11]. To avoid the problem of overfitting, a DT model with a Chi-square automatic interaction detector algorithm can be used for feature selection and classification with an accuracy rate of 74.1% [12]. The AUC value of the BC prediction model based on the fusion of the sequence forward selection algorithm and the SVM classifier can reach 0.9839 [13]. In a previous study, the LR model was used to predict BC using the same BC diagnostic dataset used in the present study, and an accuracy of 95.72% was reported [14]. Compared to the results of that study our results have less risk of overfitting and greater generalization ability due to dimensionality reduction and the XGBoost algorithm. For the early diagnosis of CVD, statistical learning and intelligent algorithms provide good support; the accuracy of SVM classification can reach 90.5% [15]. For the coronary heart disease dataset in the open database of the Framingham Heart Research Center, the AUC of the SVM algorithm can reach 0.75 [16]. Previously, XGBoost [17] was used to predict the readmission rate for patients with ischemic stroke within 90 days after discharge and achieved a final AUC value of 0.782 [18]. Among several tested algorithms, XGBoost achieved the best classification performance of the dataset of the China Acute Myocardial Infarction Registry, yielding an AUC value of 0.899 [19]. Hyperparameters have great impact on the classification performance of the XGBoost model. Therefore, in the present study, we used two datasets with large differences in BC and CVD diagnosis. The logarithmic loss of fivefold cross-validation was used to measure the performance of the model under the corresponding parameters, and the prediction performances of XGBoost, Light Gradient Boosting Machine (LightGBM), Gradient Boosting Decision Tree (GBDT), LR, RF, Back Propagation Neural Network (BPNN), and DT models were compared. Repeated sampling was performed, and the standard deviation of the results was calculated.

## Main text

### Methods

#### Dataset preprocessing

**Fig. 1:**
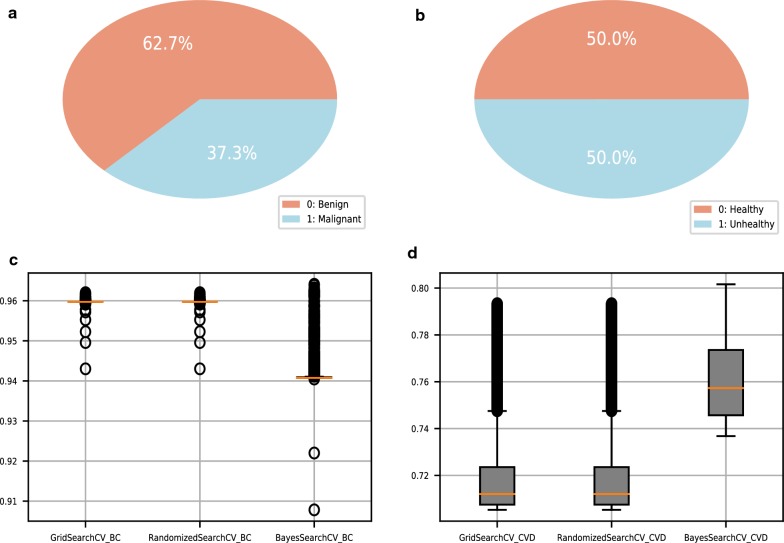
**a** Overall diagnosis in the breast cancer diagnosis dataset. **b** Overall diagnosis in the cardiovascular disease dataset. **c** Comparison of different hyperparameter optimization methods for the breast cancer dataset. **d** Comparison of different hyperparameter optimization methods for the cardiovascular disease dataset

The BC diagnosis dataset was generated at the University of California, Irvine (UCI) Machine Learning Repository with a total of 569 data points. We used the average of the 10 characteristics of the nucleus. For malignant BC tumors, the target diagnosis in the dataset is encoded “M”; for benign tumors, it is encoded “B”. For our analyses, we converted “M” to “1” and “B” to “0”. The overall dataset diagnosis results are shown in Fig. 1a. The CVD dataset was derived from Kaggle’s public dataset, which includes 65,535 patient data records and 11 characteristics. The target class “cardio” is encoded as “1” if the patient has CVD and “0” if the patient is healthy. Additionally, the IDs of patients who did not contribute to the prediction were deleted. The overall dataset diagnosis results are shown in Fig. 1b. Zero-mean normalization (Z Score) was used to process the original data. The dataset was then divided into a training set (70% of the observations) and a test set (30% of the observations). The two datasets employed in this study were both used to evaluate classifier performance, but an unbalanced structure was observed in the BC dataset (Fig. 1a). Therefore, we compared multiple indicators, including F1 score, AUC, Kolmogorov-Smirnov (KS), Receiver Operating Characteristic (ROC) curve and Precision–Recall (PR) curve, among the different models. A challenge in disease prediction is correctly evaluating whether the diseased patient becomes disease-free. In addition to comparing the performance of the classifiers, we also focused on the positive (sickness) judgment results. Because the ROC curve considers both positive and negative examples, it is suitable for evaluating the overall performance of the classifier. In comparison, the PR curve focuses only on the positive examples.

#### Models

The models, programming languages, and libraries used in this study are shown in Additional file 1. We trained all models using Python programming language (version 3.7). A personal computer with Intel (R) Core (TM) i5-7200U processor, 8 GB of RAM, and a Radeon (TM) R7 M445 GPU was used for the experiments. Each experiment required approximately 1 to 120 min to train the model.

### Results

#### Feature selection

The purpose of feature selection is to reduce the dimensions, which may improve the generalization of our algorithm [20–22]. We selected the features by analyzing the correlations among features in the BC diagnosis dataset. The correlations among radius, perimeter, and area were high, The three characteristics of compactness, concavity, and concave points were also related. Additional file 1: Table S1 illustrates the correlation among the features, and the additional doc file contains more information (see Additional file 1). Based on the correlation analysis, we considered radius and compactness as representatives. After performing feature selection in the BC diagnosis dataset, six features were retained. Considering that the correlation among the features of the CVD dataset is relatively small, the CVD dataset uses recursive feature elimination (RFE) and fivefold cross-validation to reduce the dimensions. We used the number of features corresponding to the minimal logarithmic loss. After the feature selection in the CVD dataset, nine features were retained. Additional file 1: Fig. S1 illustrates the feature selection process for the CVD dataset; the additional doc file contains more information (see Additional file 1).

#### Performance comparison of different hyperparameter optimization methods

To evaluate the effectiveness of the Bayesian parameter optimization algorithm, we used grid search and random search as comparison methods to adjust the hyperparameters of XGBoost as well as fivefold cross-validation. In this study, four hyperparameters with high influence on the XGBoost algorithm were selected for adjustment. Additional file 1: Table S2 illustrates the hyperparameter space; the additional doc file contains more information (see Additional file 1). The other parameters used the default settings, and the number of iterations was 5000. The horizontal axis of Fig. 1c, d represents different hyperparameter optimization methods in the process of hyperparameter selection, and the vertical axis represents the AUC value predicted by the XGBoost model. Fig. 1d shows that the Bayesian hyperparameter optimization method had better stability. Thus, we used the Bayesian optimization method for hyperparameter selection of all algorithms.

#### Performance comparison of the different classifiers

**Fig. 2:**
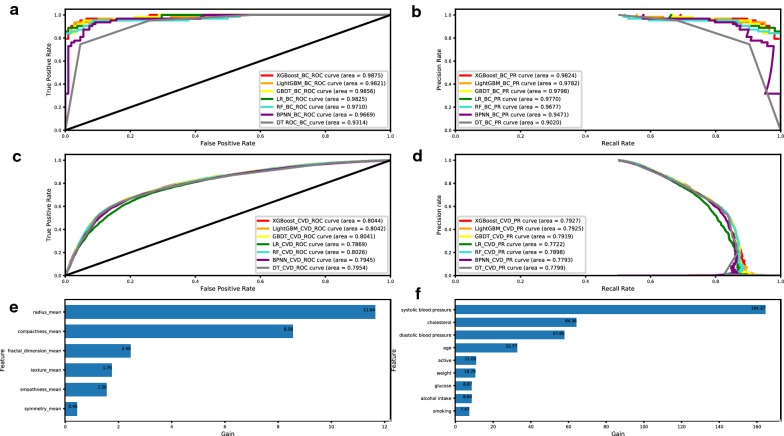
**a** ROC curves for the breast cancer diagnosis dataset. **b** PR curves for the breast cancer diagnosis dataset. **c** ROC curve for the cardiovascular disease dataset. **d** PR curves for the cardiovascular disease dataset. **e** Feature importance rankings for the breast cancer dataset. **f** Feature importance rankings for the cardiovascular disease dataset

The classification indicators of the different classifiers (LightGBM, GBDT, LR, RF, BPNN, and DT) acting on the two datasets were compared with those of the XGBoost classifier. The stability of the results was verified through 1000 repeated samplings. The mean and standard deviation of each indicator were calculated. In the small BC diagnostic dataset, XGBoost performed better than LightGBM, GBDT, LR, RF, BPNN and DT but was not as stable as GBDT. In the large CVD dataset, XGBoost’s classification performance was relatively stable (Table 1 and Fig. 2a–d).

**Table 1:** Performance indicators of different classifiers on the breast cancer diagnostic and cardiovascular disease datasets

Classifier	Indicator					
	Accuracy (%)	Precision (%)	Recall (%)	F1 score (%)	AUC	KS Value
XGBoost_BC	*94.74* (94.40,1.65)	*92.19* (93.10,3.25)	*93.65* (91.83,3.68)	*92.91* (92.38,2.29)	*0.9857* (0.9845,0.76)	0.9061
LightGBM_BC	94.74 (94.05,1.69)	92.19 (92.65,3.49)	93.65 (91.26,3.61)	92.91 (92.00,2.33)	0.9821 (0.9835,0.80)	*0.9087*
GBDT_BC	94.15 (*94.72*,1.59)	90.77 (*94.41*,3.24)	93.65 (*92.79*,2.21)	92.19 (*92.96*,2.20)	0.9856 (*0.9869*,0.66)	0.8968
LR_BC	92.40 (93.64,1.62)	89.06 (92.77,3.15)	90.48 (90.09,3.42)	89.76 (91.25,2.19)	0.9825 (0.9847,0.58)	0.8796
RF_BC	92.40 (91.81,1.94)	90.32 (90.24,3.64)	88.89 (87.77,4.67)	89.60 (88.93,2.78)	0.9710 (0.9757,0.94)	0.8690
BPNN_BC	89.47 (92.86,1.85)	89.47 (91.64,4.18)	80.95 (88.80,3.67)	85.00 (90.23,2.56)	0.9669 (0.9778,0.92)	0.8439
DT_BC	87.72 (90.75,1.83)	90.38 (86.76,3.84)	74.60 (88.53,4.92)	81.74 (87.41,2.80)	0.9314 (0.9500,1.53)	0.6997
XGBoost_CVD	73.50 (73.51,0.27)	75.80 (75.55.0.49)	69.54 (69.53,0.51)	72.54 (*72.44*,0.30)	*0.8044* (*0.8023*,0.26)	0.4733
LightGBM_CVD	73.53 (*73.56*,0.26)	75.38 (75.82,0.47)	70.40 (69.17,0.60)	72.81 (72.32,0.32)	0.8042 (0.8023,0.26)	*0.4762*
GBDT_CVD	*73.56* (73.51,0.27)	75.70 (75.60,0.49)	69.90 (69.43,0.58)	72.68 (72.38,0.33)	0.8041 (0.8023,0.25)	0.4746
LR_CVD	72.32 (71.92,0.41)	74.90 (74.02,0.72)	67.69 (67.50,0.54)	71.11 (70.62,0.40)	0.7869 (0.7829,0.38)	0.4503
RF_CVD	73.55 (73.51,0.27)	75.98 (76.02,0.72)	69.39 (68.70,0.60)	72.54 (72.17,0.32)	0.8026 (0.8012,0.26)	0.4717
BPNN_CVD	72.85 (72.81,0.31)	73.07 (73.73,1.11)	*72.96* (*70.98*,1.79)	*73.01* (72.28,0.53)	0.7945 (0.7917,0.28)	0.4686
DT_CVD	73.26 (73.12,0.17)	*76.45* (*76.79*,0.86)	67.72 (66.42,1.33)	71.83 (71.22,0.48)	0.7954 (0.7942,0.30)	0.4667

(a) Values in parentheses are the average and standard deviation of the performance indicator values. For the BC dataset, 300 samples were randomly selected from 569 samples each time and repeated 1000 times. For the CVD data set, 1000 samples were randomly selected from 65,535 samples each time and repeated 1000 times.

(b) Italics numbers indicate optimal values

#### Feature importance ranking

In this experiment, the average gain of each feature in all the trees in which it appeared was used to rank the features in importance. Features with higher values of this metric can be considered more important for prediction than features with lower values. In the BC diagnosis dataset, radius mean was the most important feature for prediction (Fig. 2e). In the CVD dataset, the patient’s systolic blood pressure was the most important feature for predictions (Fig. 2f).

### Discussion

With increasing attention being paid to computer-aided diagnosis, disease-assisted diagnosis requires reliable and interpretable classifiers. In addition to selecting a reliable classifier to achieve better prediction performance, the dataset needs to be preprocessed [23]. To improve the performance of the classifier, we compared grid search, random search, and Bayesian hyperparameter optimization methods. Unlike traditional grid search and random search methods, Bayesian parameter optimization algorithms based on Gaussian processes can find stable hyperparameters, and they are widely used in machine learning [24]. Figure 1c, d shows that compared with the smaller dataset, the larger dataset was associated with more stable Bayesian hyperparameter optimization performances. XGBoost was compared to LightGBM [25] GBDT, LR, RF, BPNN, and DT using two datasets of different sizes. Among the algorithms, XGBoost achieved the best accuracy (94.74%), precision (92.19%), sensitivity (93.65%), F1 score (92.91%), AUC (0.9875) and PR curve for the BC diagnosis dataset. However, the classification performance of GBDT was more stable over repeated sampling. For the CVD dataset, which has a large amount of data, the AUC (0.8044) and PR curve of the XGBoost algorithm were optimal, and the performance over repeated sampling was largely stable. Therefore, these findings indicate that for smaller datasets, although the XGBoost algorithm has advantages over LightGBM, GBDT, LR, RF, BPNN, and DT algorithms, it is not as stable as GBDT. For larger datasets, the XGBoost algorithm has better classification performance than the other algorithms, and its classification performance is stable (Table 1 and Fig. 2a–d). Our experiments used the gain to rank the importance of the features. A higher value of this metric indicates that the feature has higher importance for prediction. The ranking results of the feature importance are shown in Fig. 2e, f. In the BC dataset, the average value of the distance from the center of the nucleus to the edge (radius mean) of the lesion was the most important for prediction, indicating that it is important to calculate this feature of the nucleus in the digital images of FNA lesions. In the CVD dataset, the systolic blood pressure of the patient was the most important for prediction, suggesting that it is important to measure the patient’s systolic blood pressure during physical examination. Thus, the methods presented in this paper are interpretable and helpful for the predictive evaluation of disease and the identification of early, high-risk features.

## Limitations

Multiclass data can be used to compare the performance of the models used. The feature selection method needs further research. In addition to correlation analysis and RFECV, Gradient Boosted Feature Selection [26] can also be tried to further reduce relevant and non-redundant features for supervised classification problems. In future work, we would like to further improve the classification prediction performance.

## Supplementary information

**Additional file 1.** Information on feature selection, hyperparameter spaceand programing languages and libraries.The doc file contains two tables and one figure. The first table shows thecorrelation among the features of the breast cancer diagnosis dataset. Thefirst figure illustrates the feature selection process for the cardiovascular disease dataset. The second table shows the hyperparameter space.

**Additional file 2.** The dataset used in the manuscript.The .csv file contains a breast cancer diagnosis dataset and a cardiovasculardisease dataset.

## Data Availability

The datasets supporting the conclusions of this article are included within the article and its Additional file 2.

## Abbreviations

XGBoost: Extreme gradient boosting
LightGBM: Light gradient boosting machine
GBDT: Gradient boosting decision tree
LR: Logistic regression
RF: Random forest
BPNN: Back propagation neural network
DT: Decision tree
BC: Breast cancer
CVD: Cardiovascular diseases
RFE: Recursive feature elimination
ROC: Receiver operating characteristic curve
PR: Precision–recall
AUC: Area under curve
ID: Identification
FNA: Fine needle aspirate
