# Supplementary material for: Disease prediction via Bayesian hyperparameter optimization and ensemble learning
Source: BMC Res Notes. 2020 Apr 10;13:205. doi: 10.1186/s13104-020-05050-0 (PMC7146897; doi:10.1186/s13104-020-05050-0)
Supplement: Supplementary file 1 — Additional file 1. Information on feature selection, hyperparameter spaceand programing languages and libraries.The doc file contains two tables and one figure. The first table shows thecorrelation among the features of the breast cancer diagnosis dataset. Thefirst figure illustrates the feature selection process for the cardiovascular disease dataset. The second table shows the hyperparameter space. [file 13104_2020_5050_MOESM1_ESM.doc]

**Additional File 1 --- Information on feature selection, hyperparameter space and programing languages and libraries**

**Details about feature selection**

The doc file contains two tables and one figure. The first table shows the correlation among the features of the breast cancer diagnosis dataset. The first figure illustrates the feature selection process for the cardiovascular disease dataset. The second table shows the hyperparameter space.

Table S1 Feature correlation of the breast cancer diagnosis dataset

| Features | | Mean | | | | | | | | | |
| --- | --- | --- | --- | --- | --- | --- | --- | --- | --- | --- | --- |
|  |  | Radius | Texture | Perimeter | Area | Smoothness | Compactness | Concavity | Concave points | Symmetry | Fractal dimension |
| Mean | **Radius** | 1 |  |  |  |  |  |  |  |  |  |
|  | Texture | 0.323* | 1 |  |  |  |  |  |  |  |  |
|  | Perimeter | 0.997* | 0.329* | 1 |  |  |  |  |  |  |  |
|  | **Area** | 0.987* | 0.321* | 0.986* | 1 |  |  |  |  |  |  |
|  | **Smoothness** | 0.170* | -0.023 | 0.207* | 0.177* | 1 |  |  |  |  |  |
|  | **Compactness** | 0.506* | 0.236* | 0.556* | 0.498* | 0.659* | 1 |  |  |  |  |
|  | Concavity | 0.676* | 0.302* | 0.716* | 0.685* | 0.521* | 0.883* | 1 |  |  |  |
|  | Concave points | 0.822* | 0.293* | 0.850* | 0.823* | 0.553* | 0.831* | 0.921* | 1 |  |  |
|  | **Symmetry** | 0.147 | 0.074 | 0.183* | 0.151 | 0.557* | 0.602* | 0.500* | 0.462* | 1 |  |
|  | **Fractal_dimension** | -0.311* | -0.076 | -0.261* | -0.283* | 0.584* | 0.565* | 0.336* | 0.166* | 0.479* | 1 |

The remaining feature fonts are bolded. * represents significantly correlated at levels less than 0.0001.


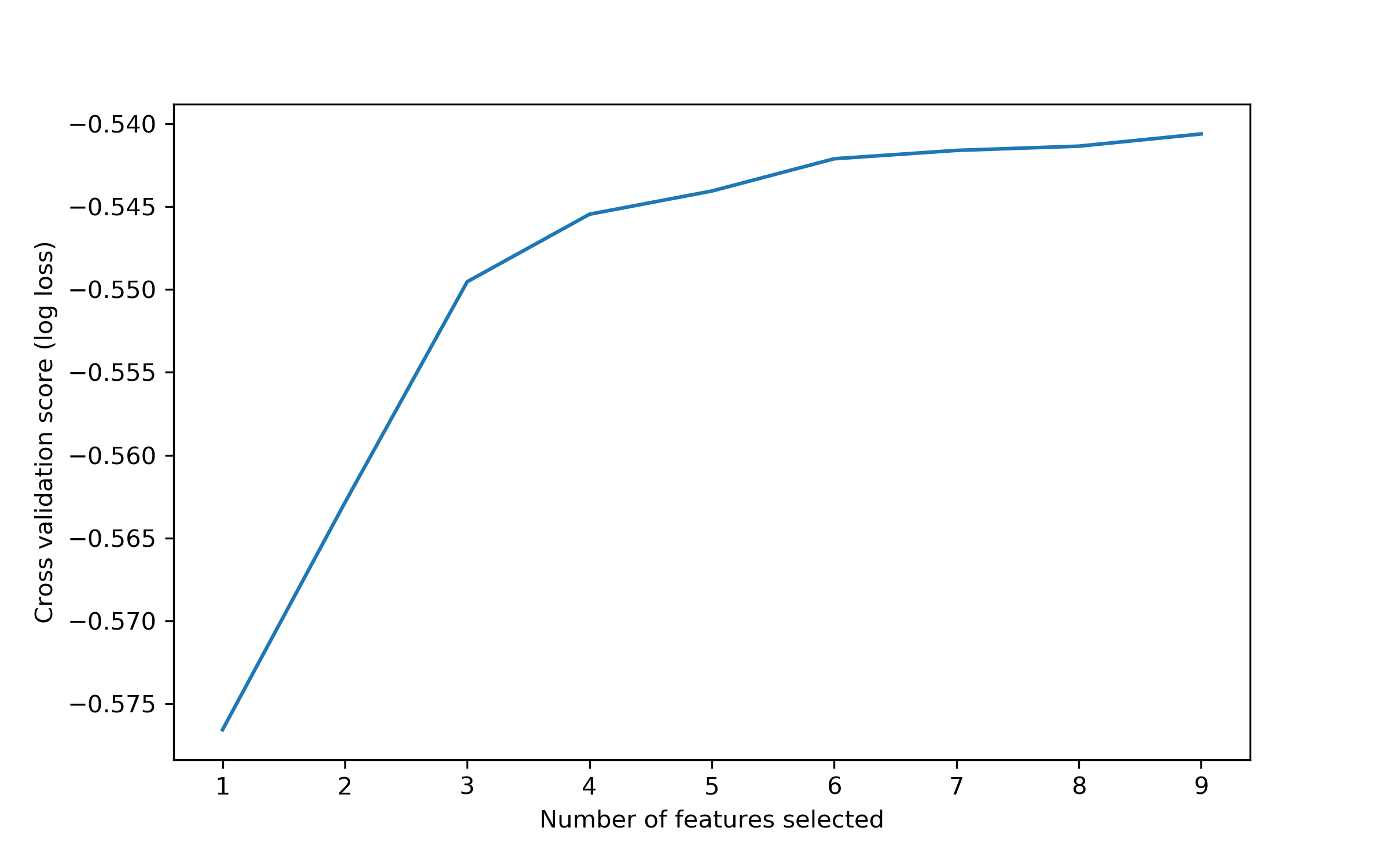


Fig. S1 Feature selection process for the cardiovascular disease dataset.

**Details about hyperparameter space**

Table S2 Hyperparameter space

| Hyperparameter | Grid Search | Randomized Search | Bayesian Search |
| --- | --- | --- | --- |
| N_Estimators | 20, 50, 100, 150, 200, 250, 300 | (20, 300) int | (20, 300) int |
| Learing_Rate | 0.01, 0.02, 0.03, ..., 0.1 | (0.01, 0.1) | (0.01, 0.1) |
| Max_Depth | 1, 2, 3, ..., 10 | (1, 10) int | (1, 10) int |
| Min_Child_Weight | 1, 2, 3, 4, 5 | (1, 5) int | (1, 5) int |

**Details about programing languages and libraries**

**Data collection**

The BC diagnosis dataset is from the UCI database (http://archive.ics.uci.edu/ml/machine-learning-databases/breast-cancer-wisconsin/). The dataset has a total of 569 data points. The mean, SE, and worst features were calculated for the 10 features of the nucleus in the digital images of the FNA breast masses. We selected the average value of the 10 features for prediction and deleted the "ID" of the patients who were not related to the prediction.

The CVD dataset is from Kaggle's publicly published dataset (https://www.kaggle.com/sulianova/cardiovascular-disease-dataset#cardio_train.csv). There were 65535 patient data records in the dataset with 11 patient characteristics, such as age, sex, systolic blood pressure, diastolic blood pressure.

**Feature selection**

For the BC diagnosis dataset and the CVD dataset, we used sklearn.preprocessing in Python (https://scikit-learn.org/dev/modules/preprocessing.html#standardization-or-mean-removal-and-variance- scaling) to perform Z-score normalization on the features and then used the statistical inference package scipy.stats (https://docs.scipy.org/doc/scipy/reference/stats.html) to calculate the Pearson correlation coefficients between the features. The multicollinearity function selects a representative feature among the features with relatively large correlation coefficients.

The CVD dataset has a large amount of data and small correlation coefficients between features, so we use the recursive feature elimination method to reduce the dimensions and specifically use the RFECV package in the sklearn library (http://lijiancheng0614.github.io/scikit-learn/ modules / generated / sklearn.feature_selection.RFECV.html) and sort the features through five-fold cross-validation to achieve dimensionality reduction.

**Hyperparameter optimization method**

Specifically, we used BayesSearchCV (http://scikit-optimize.github.io/#skopt.BayesSearchCV) in the skopt library to select a set of hyperparameters with the smallest log loss to improve the performance of the classifier.

**Classification**

Both the BC diagnosis dataset and the CVD dataset belong to the binary classification problem. The classifiers we use are XGBoost, LightGBM, GBDT, RF, BPNN and DT.

**XGBoost**

XGBoost is an integrated machine learning algorithm based on the DT algorithm proposed by Chen Tianqi. Specifically, the XGBoost Python class library (https://pypi.org/project/xgboost/) is used to predict disease.

**LightGBM**

LightGBM is a distributed gradient boosting framework based on a DT algorithm.This article uses the LightGBM package (https://lightgbm.readthedocs.io/en/latest/Python-Intro.html) from Python to make disease predictions.
